# Editorial: Interspecific hybridization in plant biology, volume II

**DOI:** 10.3389/fpls.2024.1412622

**Published:** 2024-04-26

**Authors:** Dayun Tao, Ruslan Kalendar, Andrew H. Paterson

**Affiliations:** ^1^ Yunnan Seed Laboratory/Yunnan Key Laboratory for Rice Genetic Improvement, Food Crops Research Institute, Yunnan Academy of Agricultural Sciences, Kunming, China; ^2^ National Laboratory Astana, Nazarbayev University, Astana, Kazakhstan; ^3^ Plant Genome Mapping Laboratory, University of Georgia, Athens, GA, United States

**Keywords:** interspecific hybridization, heterosis, crop improvement, synthetic polyploids, phenotypic variation

We continued with the Research Topic “Interspecific Hybridization in Plant Biology, Volume II” to discuss its fundamental and applied consequences. While the topic was open to salient work in other taxa, published work all addressed plants, in which such work is prominent. A critical threshold for publication was the extent to which findings are of cross-cutting interest and importance, i.e., not only to those working on the target taxon but to a wide range of biological scientists (Tao et al., 2022).

## Interspecific hybridization and genome dominance

Interspecific hybridization, a significant evolutionary phenomenon, involves fusing two distinct genomes, often leading to whole-genome duplication and the generation of genetic novelty that serves as raw material for selection and evolution (Carscadden et al., 2023; Deb et al., 2023). While the overwhelming majority of interspecific hybrids quickly go extinct, in rare cases this process can increase the adaptive potential of hybrid individuals through newly generated genomic diversity, but it can also induce genetic and epigenetic changes in response to genomic instability (Fang et al., 2024).

Genome dominance refers to hybrids in which one parent genome becomes ‘dominant’ (Kan et al., 2024), as manifested by physical replacement of chromatin from the subordinate genome and/or skewed gene expression. Nucleolar dominance, the functional expression of only one parental set of ribosomal genes in hybrids, is another example of an intragenomic competitive process involving only ribosomal DNA. There is evidence that nucleolar dominance in plants is difficult to predict, independent of maternal effect or parental ribosomal DNA copy number, reversible, developmentally regulated, and dosage dependent. All this makes nucleolar dominance a challenging phenomenon to study. Despite the wealth of information describing gene silencing mechanisms, the most intriguing aspect of nucleolar dominance, the molecular basis that dictates which genes are silenced and which are transcribed, remains to be seen. Most hybrids and allopolyploids exhibit some degree of genome dominance. An example of genome dominance in allopolyploids involving both of the above mechanisms is observed in ×*Festulolium* (hereafter *Festulolium*), a hybrid genus between *Festuca* (fescue) and *Lolium* (ryegrass). Research by Mahelka et al. investigated this phenomenon in detail, observing the preferential elimination of *Festuca* chromosomes in male meiosis, which was probably linked to the silencing of the *Festuca* alleles of two kinetochore genes before and during meiotic division. This silencing probably resulted from a change in the spatial architecture of the hybrid nucleus during the cell cycle, as genes located over more than half of *Festuca* chromosome 7 were more or less silenced before and during meiosis but not in mitosis. Thus, different levels of genome dominance could be determined by various factors without these phenomena being interdependent. This study provides new evidence that nucleolar dominance is established early after genome merging in interspecific hybridization and is already complete in the F_2_ generation, that it is independent of maternity, and that it is consistent with genome dominance at both the chromosome and transcriptome levels. The dominant genome is always the same (*Lolium* in *Lolium* × *Festuca* hybrids and *F. pratensis* in *F. glaucescens* × *F. pratensis* hybrids).

## Interspecific hybridization for improvement of genetic variability

The genus *Camelina* has attracted the attention of researchers because of one of its outstanding representatives, *Camelina sativa* (L.) Crantz, or false flax. This oilseed crop has become a platform for various genetic engineering studies. Cultivated *Camelina* species have evolved through a series of polyploidization events, which have acted as bottlenecks limiting the species’ genetic diversity. The genetic paucity of *C. sativa* is considered to be the main limitation to successful breeding and improvement of this crop. A potential solution to this challenge could be gene introgression from wild *Camelina* species or resynthesized allohexaploid *C. sativa*. Blume et al. what is arguably the most complete integrated evolutionary model for the genus *Camelina* based on recently described findings, which allows efficient improvement of *C. sativa* through interspecific hybridization with its wild relatives. Allohexaploid *C. sativa* hybridizes poorly with diploid species and more efficiently with tetraploids or complex allopolyploids. The most promising approach is the hybridization of *C. sativa* with its closest relative, *C. macrocarpa*. However, the cytotype identity of the wild relative should be considered, as *C. macrocarpa* type 2 has a different genome organization explained by its evolutionary history. Finally, considering the evolutionary origin of *C. sativa*, a pathway for the resynthesis of this allohexaploid crop was proposed. Such synthetic *C. sativa* could be used for gene introgression from the diploid *C. hispida*, which does not hybridize with *C. sativa*, or from the tetraploid *C. microcarpa* (*C. intermedia*), with which hybridization has had limited success.

The common bean (*Phaseolus vulgaris* L.) is Latin America and Africa’s most widely consumed legume. The reproductive development of domesticated common beans is particularly susceptible to high-temperature stress, with day and night temperatures exceeding 30°C and 20°C, respectively, resulting in significant yield losses. The wild common bean is organized into two geographically isolated and genetically distinct wild gene pools (Mesoamerican and Andean), diverging from a common ancestor domesticated as the common bean in Mexico and South America. Due to its natural adaptation to arid conditions, the desert tepary bean (*Phaseolus acutifolius* A. Gray) is a promising source of adaptive genes. Hybridization between the two species is challenging, requiring *in vitro* embryo rescue and multiple cycles of backcrossing to restore fertility. This labor-intensive process limits the development of mapping populations needed to study heat tolerance. Cruz et al. demonstrate the development of an interspecific mapping population using a novel technique based on a bridging genotype derived from *P. vulgaris*, *P. acutifolius* and *P. parvifolius*. The population was based on two wild *P. acutifolius* accessions repeatedly crossed with elite Mesoamerican common bean breeding lines and contained 59.8% introgression from wild tepary but also genetic regions from *P. parvifolius*, a relative represented in some early bridging crosses. It was shown that the bridging genotype could interbreed common bean with tepary and positively influence the physiology of the derived interspecific lines, which showed beneficial variation for heat tolerance.

Global consumption of domesticated Asian rice, *Oryza sativa*, is increasing every year, and to keep pace with demand, annual increases in rice yield must be increased. The non-domesticated rice relative, *O. longistaminata*, is a valuable genetic resource for improving the domesticated Asian rice, *O. sativa*. As a perennial, cross-pollinated species native to sub-Saharan Africa, *O. longistaminata* is highly diverse. The frequency and extent of such natural introgressions and their impact on the evolution of *O. longistaminata* have not been investigated. As hybridization with *O. sativa* is complicated by significant breeding barriers (e.g., endosperm abortion, F_1_ sterility, and hybrid breakdown), manipulating the crossability between these two species could accelerate introgression efforts. Labroo et al. investigated the conservation, management, and use of *O. longistaminata* germplasm to quantify the population structure and diversity of this species across its geographic range, which includes most of sub-Saharan Africa, and to determine phylogenetic relationships with other related rice species in Africa, including the prevalence of interspecific hybridization between *O. longistaminata* and *O. sativa*. This study includes *O. longistaminata* accessions together with *O. sativa*, *O. barthii* and *O. glaberrima* control outgroups and control interspecific *O. sativa/O. longistaminata* hybrids. Three genetic subpopulations of *O. longistaminata* were identified, corresponding geographically to Northwest Africa, Pan-Africa and Southern Africa. It was confirmed that, perhaps counter-intuitively, *O. longistaminata* is more closely related to the Asian species *O. sativa* than to the African species *O. barthii* and *O. glaberrima*. Recent introgression between *O. sativa* and *O. longistaminata* has been bidirectional. Furthermore, low levels of *O. sativa* alleles admixed in many predominantly *O. longistaminata* accessions suggest that introgression also occurred in the distant past, but only in southern Africa.

## Hybrid breeding and heterosis

Hybrid breeding exploits dominance effects by breeding for inbred parents whose F_1_ progeny will have positive heterosis (Kakoulidou and Johannes, 2023; Legarra et al., 2023). To evaluate and select for heterosis, hybrid breeding typically uses self-pollinated or double-haploid inbred lines, followed by progeny evaluation in heterotic pools (Labroo et al., 2021). As a representative approach, reciprocal recurrent selection has been developed to assist in developing selective recombinant maize lines with heterosis selection. It is a cyclic breeding procedure designed to improve the crossing of two populations from different heterotic groups, in which genotypes from two homozygous populations are evaluated in reciprocal crosses, and the best-adapted genotypes from each population are selected and recombined to produce an improved hybrid. To assess the effectiveness of competing decision strategies, Zhang and Wang present a modular simulation framework for reciprocal recurrent selection-based hybrid breeding. Consisting of several modules such as heterotic separation, genomic prediction, and genomic selection, this simulation framework allows breeders to efficiently simulate the hybrid breeding process with different simulator options and decision strategies. The framework also incorporates a broad sense of heritability as an adjustment for environmental effects to bring it closer to reality. A sensitivity analysis of the environmental effect was performed for both the phenotypic predictor and the Bayesian predictor, which are the two predictors that would be affected by the varying phenotypic values. An important finding was that even with imperfect genetic prediction results, genomic selection, and mating strategies would benefit hybrid breeding.

Potted miniature rose varieties are introduced to the market every year, but breeding studies take a long time and are labor-intensive and costly. Meral has studied the high fruiting rate, many seeds per fruit, and low germination rate; few offspring have been obtained. At the same time, these genotypes had dwarf and large flower-diameter plants. In the combinations where *Rosa centifolia* was used as a pollen parent, fragrant plants were obtained, indicating that Rose Bling Love Star and Rose White Star can be used as seed parents, as evidenced by the high fruit set and number of seeds per fruit. In addition, qualitative and quantitative analysis of the progeny and calculations of heterosis and heterobeltiosis have proved to be valuable tools for evaluating the performance of the progeny on the parents, thus facilitating the selection of candidate varieties with better-performing traits. As the cross combinations can be determined using the parental performance information that is now available, this study is expected to contribute to the breeding success of miniature roses. The combinations chosen this way are more likely to be successful than those chosen randomly. Breeders can quickly adopt the practice of selecting their cross combinations and parents more carefully to increase the effectiveness of their breeding programs and bring more new types to the market.

## Recombination and speciation

Understanding how new species arise and reproductive isolation evolves is an important, long-standing topic in evolutionary biology. Recombination has long been hypothesized to play an essential role in determining the rate of speciation, hybridization, and adaptation (Ortiz-Barrientos et al., 2016; Feulner and De-Kayne, 2017; Ortiz-Barrientos and James, 2017). Heterogeneity in local recombination rate underlies many observed patterns across the genome (e.g., actively recombining regions are typically gene-rich and depleted of repetitive DNA) and can strongly influence the permeability of genomic regions to interspecific introgression. The larger the region lacking recombination, the greater the likelihood that species incompatibility gene(s) will be present in that region, rendering the entire non- or infrequently-recombining block impermeable to interspecific introgression. Large plant genomes tend to have a highly heterogeneous recombination landscape, with recombination often occurring predominantly at the ends of chromosomes and rarely in central regions. There is strong evidence that infrequently recombining genomic regions show higher species differentiation than actively recombining regions, suggesting that the former play an essential role in speciation and contribute to limiting gene flow between hybridizing taxa. However, the role of infrequently recombining regions in speciation has yet to be fully understood. Rarely recombining regions may play an essential role in maintaining species identity in actively hybridizing species. Wong and Filatov review the relationship between recombination and introgression in plants and argue that large, rarely recombining regions are likely to play an essential role in maintaining species identity in actively hybridizing plant species. Further evidence of enrichment of rarely recombining regions for genes responsible for species-specific traits is needed to confirm their specific role in maintaining species identity in the face of interspecific gene flow. Furthermore, reduced recombination may not be the cause but rather the result of diversifying selection during speciation with gene flow, or in other words, selection for alleles responsible for local adaptation.

In summary, this Research Topic has increased knowledge of interspecific hybridization, hybrid breeding, and heterosis to increase genetic variability in plant genetics and evolution. On some topics, such as genomics of interspecific hybrid heterosis, transmission genetics across species boundaries, genomic responses to interspecific hybridization, and genomics of natural or artificial polyploid formation, this platform for exchange and progress shows important potential for further advances.

## Author contributions

DT: Conceptualization, Funding acquisition, Investigation, Writing – original draft, Writing – review & editing. RK: Conceptualization, Funding acquisition, Investigation, Writing – original draft, Writing – review & editing. AP: Conceptualization, Funding acquisition, Investigation, Writing – original draft, Writing – review & editing.
